# Part I: Accuracy of Teledermatology in Inflammatory Dermatoses

**DOI:** 10.3389/fmed.2020.585792

**Published:** 2020-10-27

**Authors:** Mara Giavina-Bianchi, Raquel Sousa, Eduardo Cordioli

**Affiliations:** Department of Telemedicine, Hospital Israelita Albert Einstein, São Paulo, Brazil

**Keywords:** teledermatology, accuracy, telemedicine, inflammatory dermatoses, telehealth

## Abstract

Teledermatology is assuming a progressively greater role as a healthcare delivery method, especially now, during this pandemic time. It is important to know how accurate this tool is for different skin diseases. Most of the studies have focused on skin neoplasms or general dermatology. Studies based on a large number of inflammatory dermatoses have not yet been performed. Such knowledge can help dermatologists to decide whether endorsing this method or not. Our objective was to determine the accuracy of teledermatology in inflammatory dermatoses in a robust number of cases. A retrospective cohort study was conducted in São Paulo, Brazil, from July 2017–18, where a store-and-forward Teledermatology project was implemented under primary-care attention to triage surgical, more complex, or severe dermatoses. A total of 30,976 patients presenting 55,012 lesions took part in the project. Thirteen participating teledermatologists had three options to refer the patients: directly to biopsy, to the in-person dermatologist or back to the general physician with most probable diagnosis and management. In the group referred to the in-person dermatologist, we looked for the 20 most frequent International Classification of Diseases and Related Health Problems- 10th revision (ICD-10) of inflammatory dermatoses, which resulted in 739 patients and 739 lesions. As patients had been triaged by teledermatology previously, we were able to compare ICD-10 codes filled both by teledermatogists and by in-person dermatologists. The proportion of complete, partial, and no agreement rates between the in-person dermatologist's and the teledermatologist's diagnoses was used for accuracy. We also calculated Cohen's kappa, a statistical measure of inter-rater agreement, for complete agreement. The mean complete agreement rate for all twenty dermatoses was 78% (31–100%) and kappa = 0.743; partial agreement 8%; and no agreement 14%, presenting variability according to the disease. Our study showed that teledermatology for inflammatory dermatoses has a high accuracy. This result reassures that it can be a proper option for patient care.

## Introduction

Telemedicine, especially in this pandemic moment, is of great value for delivering healthcare. It has the potential to improve access to subspecialty expertise, reduce healthcare costs, and improve the overall quality of care. Dermatology is particularly suitable for this care system. The three main teledermatology delivery platforms are: synchronous (RT: real-time teledermatology), asynchronous (SF-TD), and hybrid (both synchronous and asynchronous forms). Synchronous teledermatology employs live video conferencing between the patient and the teledermatologist. Asynchronous teledermatology is a method whereby clinical or dermoscopy dermatologic images are obtained, sent to the responding dermatologist, who can review them at later time. Although it provides high-resolution dermatologic images and enables an efficient practice that can be performed across time zones, this modality is limited by the ability of the teledermatologist to obtain additional clinical history while evaluating the case (1).

Rates of diagnostic accuracy by teledermatology vary from study to study; the majority have found rates to be in the range of 75–80%, comparable to those with in-person care (1). Nevertheless, most of the studies were focused on skin neoplasms, especially skin cancer and pigmented lesions (2–5), or on general dermatology (6–10). A recent systematic review concluded that robust implementation studies of teledermatology are needed, with attention to reducing risk of bias when assessing diagnostic accuracy (2). For this reason, we performed a study with the aim of determining the accuracy of teledermatology for inflammatory dermatoses in a robust number of cases, assessing the agreement rate between the in-person dermatologist's and the teledermatologist's diagnoses.

## Materials and Methods

This was a retrospective cohort study designed to assess concordance between diagnoses made by in-person dermatologists and teledermatologists, approved by the Ethics Committee of Hospital Israelita Albert Einstein (CAAE: 97126618.6.0000.0071). We analyzed the reports of 30,976 patients included in a teledermatology triage project conducted in the city of São Paulo, Brazil, from July 2017 to July 2018.

### Teledermatology Triage Project

**Figure 1:**
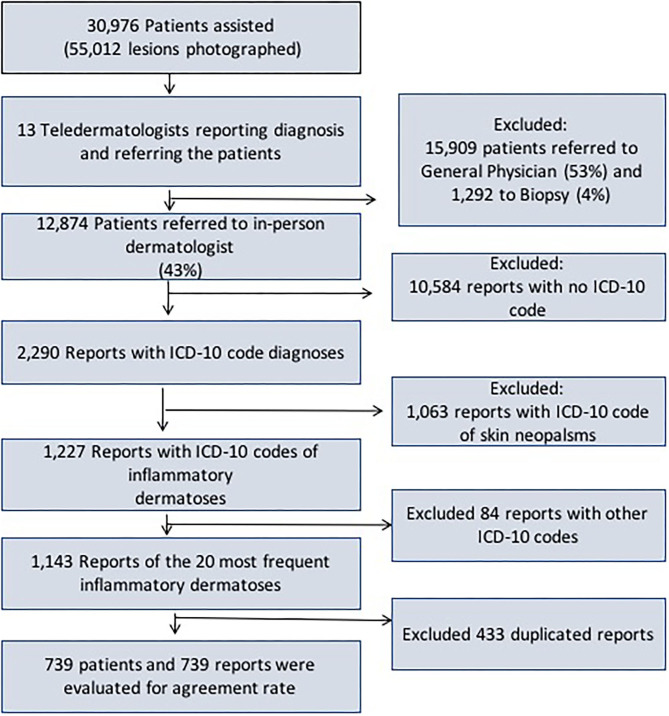
Frequency of patients included, photographed lesions and referrals made by the teledermatologists, along with the flow used to select the reports to assess the diagnosis accuracy.

Since there was a long patients' waiting list for an appointment with a dermatologist in the public health service, the aim of the teledermatology project was to triage the patients, in such a way that the severe, more complex, or surgical cases would be prioritized for biopsy procedure and in-person dermatologists, and the mild cases would be managed in the primary care attention along with the general physician (GP). Briefly, there were 57,832 patients under public primary-care attention who were on a waiting list for an appointment with a dermatologist, after being referred by the primary care physician. All of them were consecutively phoned to go to one of the three public municipal hospitals. Once there, their demographic data, a short clinical history and photographs of their skin lesions were taken by a nurse or a health technician, utilizing a cell phone app created for this purpose. Thirty thousand nine hundred seventy-six individuals responded to the call and attended the project. All their data and images were securely uploaded to a platform accessed by thirteen teledermatologists from Hospital Albert Einstein, authorized to do so through login and password at a later time (store-and-forward telemedicine). The thirteen dermatologists were Brazilian Board-certified to decrease the chance of diagnostic error. Once logged, the teledermatologists evaluated the cases, and they had to elaborate the most probable diagnosis and management. Next, they had to decide among three options to refer the patients: (1) directly to biopsy (with subsequent follow-up with an in-person dermatologist), (2) to an in-person dermatologist and (3) back to the general physician who had referred him/her to the dermatologist in the first place. Figure 1 shows the frequency of patients included, photographed lesions, and referrals made by the teledermatologists, along with the flow used to select the reports to assess the accuracy. Teledermatologists were Hospital Israelita Albert Einstein employees (a private institution), and in-person dermatologists were public health service employees.

### Study Design

We selected only the group referred to in-person dermatologist (12,874). Then, we assessed the reports that had the International Code of Diseases diagnoses filled by the in-person dermatologists (2,290). Next, we separated the reports filled with ICD-10 codes of inflammatory dermatoses (1,227). Afterwards, we looked for the 20 most frequent dermatoses to include in our study (1,143). As the last step, we eliminated duplicity of reports from the same patient, totalizing 739 reports from 739 patients (Figure 1).

We classified the rate of agreement as: (1) complete agreement when ICD-10 code used in both reports were the same, (2) partial agreement when ICD-10 used in both reports were different, but in the same group of disease (Table 1), posing as a probable differential diagnosis, and (3) no agreement when both reports did not fill the previous two conditions. As many inflammatory diagnoses are based on clinical diagnosis, we considered in-person dermatologist diagnosis as our gold standard diagnosis. For this reason, the rate of agreement between in-person dermatologists and teledermatologists was stated in this research as accuracy.

**Table 1:** Group of skin disorders and respective dermatoses present in this study considered as partial agreement between in-person dermatologists' and teledermatogists' diagnosis.

**Group of disorder**	**Dermatoses**
Skin adnexal glands (sebaceous, sweat)	Acne, rosacea, hidradenitis
Skin Hyperpigmentation	Chloasma, post-inflammatory hyperpigmentation
Erythematous-scaly patches	Psoriasis, dermatophytosis, pityriasis versicolor, actinic keratosis, seborrheic dermatitis, lupus erythematosus
Skin Hypopigmentation	Vitiligo, leukoderma
Eczematous	Atopic dermatitis, contact dermatitis, dyshidrosis, stasis dermatitis, nummular dermatitis, xerosis, lichen simplex chronicus, pityriais alba, urticaria, photoallergy, seborrheic dermatitis
Alopecia	Androgenetic alopecia, alopecia areata, telogen effluvium, cicatricial alopecia

### Statistical Analysis

Rates of concordance were expressed using percentages and Cohen's kappa coefficient, which was used to compare between groups of inter-rater observers (Graph Pad Prism 6.0). The guidelines first created by Landis and Koch (11) used to characterize kappa values are as follows: kappa < 0: no agreement, 0.00–0.20: slight agreement, 0.21–0.40: fair agreement, 0.41–0.60: moderate agreement, 0.61–0.8: substantial agreement, and 0.81–1.00: almost perfect agreement.

## Results

Table 2 shows the 26 most frequent inflammatory dermatoses diagnosed by teledermatology according to number of patients, lesions, and sex distribution. This constitutes 78% (24,210/30,976) of the total number of patients and 50% (27,519/55,012) of the lesions diagnosed in the overall teledermatology project. The female and male participation was 70 and 30%, respectively, although the female population accounts for 52.6% in the city of São Paulo (12). The mean number of inflammatory dermatosis lesion per patient was 1.1.

**Table 2:** Most frequent inflammatory dermatoses diagnosed by teledermatologists according to number of patients, sex distribution, and number of photographed lesions.

**ICD-10 code**	**Dermatosis**	**Patients (*n*)**	**Male (*n*)**	**Female (*n*)**	**Photographed lesions (*n*)**
B35	Dermatophytosis	3,064	843	2,221	3,496
L70	Acne	2,662	851	1,811	3,217
L81.1	Chloasma	1,749	121	1,628	1,840
L20.9	Atopic dermatitis	1,648	603	1,045	2,058
L85.3	Xerosis	1,632	478	1,154	1,815
L57.9	Solar lentigo	1,584	223	1,361	1,870
L21.9	Seborrheic dermatitis	1,259	426	833	1,421
L23/L24/L25	Contact dermatitis	1,203	325	878	1,333
L81.0	Post-inflammatory hyperpigmentation	1,139	256	883	1,303
L81.5	Leukoderma	1,029	829	200	1,102
L65	Telogen effluvium	881	20	861	891
L30.5	Pityriasis alba	796	282	514	881
L64.9	Androgenetic alopecia	765	135	630	774
B36.0	Pityriasis versicolor	727	264	463	862
L60	Nail disorders	586	94	492	629
L40.0	Psoriasis	551	249	302	760
L80	Vitiligo	550	228	322	694
B08.1	Molluscum contagiosum	366	168	198	423
L30.1	Dyshidrosis	358	84	274	387
L28	Lichen simplex chronicus	340	157	183	368
L63	Alopecia areata	331	135	196	348
L71	Rosacea	248	60	198	257
I83.1	Stasis dermatitis	202	92	110	209
L30.0	Nummular dermatitis	201	59	142	218
L74.5	Focal Hyperhidrosis	200	86	114	209
L50	Urticaria	139	40	99	154
Total		24,210	7,108	17,112	27,519

Table 3 assesses the 20 most frequent inflammatory dermatoses diagnosed by in-person dermatologists of which we were able to recover the ICD-10 codes, along with the accuracy of teledermatology found in our study. The mean frequency of complete agreement was 78% for all 20 dermatoses tested (573/739) and its kappa coefficient was 0.743, which is considered a substantial agreement. Xerosis had the lowest rate (31%; kappa = 0.173) and psoriasis and focal hyperhidrosis showed the greatest (100%; kappa = 1.00). A partial agreement was verified in 8% of all cases (60/739), ranging from 0% (dermatophytosis, atopic dermatosis, molluscum, psoriasis, pityriasis versicolor, and focal hyperhidrosis) to 46% (xerosis). No agreement was found in 14% (106/739); psoriasis and focal hyperhidrosis with the lowest rate (0%) and pityriasis versicolor with the highest (44%).

**Table 3:** Most frequent inflammatory dermatosis diagnosed by in-person dermatologists and agreement with teledermatology diagnoses.

**Diagnoses(*n* =)**	**Complete agreement *n* (%)**	**Complete agreement kappa**	**Partial agreement *n* (%)**	**No agreement *n* (%)**
Acne (122)	113 (93)	0.924	1 (1)	8 (6)
Dermatophytosis (83)	52 (63)	0.564	0 (0)	31 (37)
Atopic Dermatitis (81)	75 (93)	0.923	0 (0)	6 (7)
Post-inflammatory hyperpigmentation (59)	20 (34)	0.206	16 (27)	23 (39)
Contact dermatitis (52)	40 (77)	0.743	5 (10)	7 (13)
Androgenetic alopecia (49)	38 (78)	0.751	8 (16)	3 (6)
Chloasma (36)	32 (89)	0.883	2 (5.5)	2 (5.5)
Molluscum contagiosum (36)	34 (94)	0.943	0 (0)	2 (6)
Vitiligo (30)	27 (90)	0.895	1 (3)	2 (7)
Seborrheic dermatitis (29)	10 (34)	0.213	13 (45)	6 (21)
Psoriasis (26)	26 (100)	1.000	0 (0)	0 (0)
Telogen effluvium (24)	23 (96)	0.957	1 (4)	0 (0)
Alopecia areata (21)	20 (95)	0.951	1 (5)	0 (0)
Rosacea (20)	17 (85)	0.839	1 (5)	2 (10)
Pityriasis versicolor (16)	9 (56)	0.481	0 (0)	7 (44)
Nail disorders (14)	12 (86)	0.847	0 (0)	2 (14)
Focal hyperhidrosis (11)	11 (100)	1.000	0 (0)	0 (0)
Xerosis (13)	4 (31)	0.173	6 (46)	3 (23)
Urticaria (9)	7 (78)	0.754	2 (22)	0 (0)
Nummular dermatitis (8)	3 (37.5)	0.247	3 (37.5)	2 (25)
Total (739)	573 (78)	0.743	60 (8)	106 (14)

Partial agreement frequency and the description of each inflammatory dermatoses are shown in Table 4. Post-inflammatory hyperpigmentation reached the highest number, with 16 cases, all of them with the same diagnosis. On the other hand, seborrheic dermatitis had 13 cases of partial agreement with 11 different diagnoses.

**Table 4:** Differences between teledermatogists' and in-person dermatologists' diagnosis in cases considered partial agreement for inflammatory dermatosis.

**Teledermatologist diagnosis**	**Partial agreement (*n*)**	**In-person diagnosis**
Acne	1	1 hidradenitis
Rosacea	1	1 acne
Contact dermatitis	5	4 atopic dermatitis, 1 lichen simplex chronicus
Nummular dermatitis	3	2 pitiryasis alba, 1 dyshidrosis
Urticaria	2	1 atopic dermatitis, 1 contact dermatitis
Xerosis	6	4 atopic dermatitis, 1 pityriasis alba; 1 photoallergy
Seborrheic dermatitis	13	3 androgenic alopecia, 1 contact dermatitis, 1 psoriasis, 1 dermatophytosis, 1 rosacea, 1 hidradenitis suppurativa, 1 pityriasis alba, 1 phototoxic allergy, 1 perifolicullitis capitis, 1 lupus erythematosus, 1 actinic keratosis
Vitiligo	1	1 leukoderma
Post-inflammatory hyperpigmentation	16	16 chloasma
Chloasma	2	2 post-inflammatory hyperpigmentation
Androgenetic alopecia	8	4 alopecia areata, 3 telogen effluvium, 1 cicatricial alopecia
Alopecia areata	1	1 androgenetic alopecia
Telogen effluvium	1	1 androgenetic alopecia

## Discussion

The differences in frequency for the inflammatory dermatoses between Tables 2 and 3 are due to the fact that although one disease could be very frequently diagnosed by the teledermatologists (Table 2), but it could not be referred as frequently to the in-person dermatologists (Table 3). That, in fact, was the reason for solar lentigo, leukoderma, pityriasis alba, dyshidrosis, lichen simplex chronicus and stasis dermatitis to be left out of the second table. These dermatoses were mostly referred back to the GP along with the diagnosis/management, and they were present only in few cases for the in-person dermatologist, not included in the 20 most frequent ones as the inclusion criteria.

This fact is also very relevant when discussing accuracy. Since the aim of the teledermatology triage project was to prioritize the severe, more complex, or surgical cases for biopsy and in-person dermatologists and to manage the mild cases under the primary-care attention along with the GP, the inflammatory disorders diagnosed by in-person dermatologists in Table 2 have a potential bias of being the most challenging cases. Typical or “regular” inflammatory dermatoses were most likely diagnosed and referred to the GP. Therefore, the rate of agreement found would be probably even higher if the more typical cases were analyzed. The results of our study showed a high agreement rate between diagnoses made by teledermatologists and in-person dermatologists, corroborating the idea that teledermatology is accurate for inflammatory dermatoses. If we add the total (78%) and partial (8%) agreement rates, we will achieve 86% (kappa = 0.846), which is considered an almost perfect agreement, and only 14% of no agreement. This would be remarkable even if we were not discussing the potential bias above. According to literature, SF-TD had an accuracy in general of 64 and 65% in medium size studies (*n* = 109 and 163, respectively) and 90 and 95% in small size studies (*n* = 50 and 10, respectively) (6). Another article found an agreement of 90% in 120 cases of SF-TD consultations (7). Lim et al. reported 88% agreement in 53 cases (8). Weingast et al. evaluated 263 patients and found accuracy of 80% (9). O'Connor et al. assessed 40 patients and encountered accuracy of 83% (10).

There were different accuracies among the 20 most frequent dermatoses diagnosed by in-person dermatologists in our study. Eight diseases reached very high complete agreement rate, 90% or above: acne, atopic dermatitis, molluscum contagiosum, vitiligo, psoriasis, telogen effluvium, alopecia areata, and focal hyperhidrosis. Six disorders showed a good total agreement rate, 70–89%: contact dermatitis, androgenetic alopecia, chloasma, rosacea, nail disorders, and urticaria. Six inflammatory dermatoses were less accurate (<60% of total agreement): dermatophytosis, post-inflammatory hyperpigmentation, seborrheic dermatitis, pityriasis versicolor, xerosis, and nummular dermatitis. What were those diseases mistaken for? In order to verify that, we checked if they could be classified as possible differential diagnosis, which we considered to be a partial agreement.

Examining the six diseases with total agreement ranging from 70–89% and considering the partial agreement rate, three of them would have a considerable change. Contact dermatitis would increase from 77 to 87%, due to five cases that were in fact four atopic dermatitis and one lichen simplex chronicus. Androgenetic alopecia (AGA) would increase from 78 to 94%, due to four alopecia areata, three telogen effluvium and one cicatricial alopecia. Chloasma would also raise from 89 to 94.5% accuracy if we included the two cases of post-inflammatory hyperpigmentation in Table 4.

Most interestingly, anyhow, is to look for the diseases showing the least accuracy. Post-inflammatory hyperpigmentation (PIH) should have a separate interpretation once all 16 cases of partial agreement had the same diagnosis: chloasma. One hypothesis is a typing error, because their ICD-10 codes are almost the same (L81.0 and L81.1). Another one is that dermatologists misuse chloasma ICD-10 code for PIH, since chloasma is a very frequent disease and its ICD-10 code may be already known by heart while PIH code would not. Nummular dermatitis and xerosis would at least double the accuracy (37.5–75% and 31–77%, respectively) if we considered the partial agreement diagnosis. Seborrheic dermatitis (SD) was the disease with the highest number of differential diagnosis (13) and considering the partial agreement rate, accuracy would go from 34 to 79%. On the scalp, SD was diagnosed as AGA and perifolicullitis capitis, which could even occur simultaneously, and on the skin, could be confused with many diseases such as psoriasis, eczemas, lupus erythematous, dermatophytosis, rosacea, and actinic keratosis. Dermatophytosis and pityriasis versicolor were the least accurate diagnosis when total and partial agreement were considered, 63 and 56%, respectively. This may be due to some limitation in assessing the lesions through teledermatology or to a great variety of possible differential diagnoses. Again, in our study, the fact that the most typical cases were meant to be treated by teledermatology and not sent to in-person dermatologists could have played an important role in these two dermatoses.

Although this was a retrospective study and much data was missing, we believe this was one of the studies with the largest number of inflammatory diseases included in the literature. The study was performed in two centers and different dermatologists performed the tele and in-person examinations. This is beneficial, in a way that we assessed the agreement between different examiners, but it also may have some bias, since the technical skills in the two groups may be different.

Our study in a large number of patients presenting the most common inflammatory dermatoses showed that the mean accuracy of teledermatology was high, varying according to the disease. This result reassures that store-and-forward teledermatology is as proper option for patient care.

## Data Availability Statement

The raw data supporting the conclusions of this article will be made available by the authors, without undue reservation.

## Ethics Statement

The studies involving human participants were reviewed and approved by Ethics Committee of Hospital Israelita Albert Einstein (CAAE: 97126618.6.0000.0071). Written informed consent from the participants' legal guardian/next of kin was not required to participate in this study in accordance with the national legislation and the institutional requirements.

## Author Contributions

MG-B, RS, and EC were responsible for the study design. MG-B was responsible for writing the article. MG-B and RS were responsible for data analyzes. All authors contributed to the article and approved the submitted version.

## Conflict of Interest

The authors declare that the research was conducted in the absence of any commercial or financial relationships that could be construed as a potential conflict of interest.
